# Checkpoints to the Brain: Directing Myeloid Cell Migration to the Central Nervous System

**DOI:** 10.3390/ijms17122030

**Published:** 2016-12-02

**Authors:** Meredith Harrison-Brown, Guo-Jun Liu, Richard Banati

**Affiliations:** 1 Discipline of Medical Imaging & Radiation Sciences, Faculty of Health Sciences, The University of Sydney, Sydney, NSW 2141, Australia; gdl@ansto.gov.au (G.-J.L.); richard.banati@sydney.edu.au (R.B.); 2 Australian Nuclear Science and Technology Organisation, Sydney, NSW 2234, Australia; 3 Brain and Mind Centre, The University of Sydney, Sydney, NSW 2006, Australia

**Keywords:** myeloid cell, brain, transplantation, microglia

## Abstract

Myeloid cells are a unique subset of leukocytes with a diverse array of functions within the central nervous system during health and disease. Advances in understanding of the unique properties of these cells have inspired interest in their use as delivery vehicles for therapeutic genes, proteins, and drugs, or as “assistants” in the clean-up of aggregated proteins and other molecules when existing drainage systems are no longer adequate. The trafficking of myeloid cells from the periphery to the central nervous system is subject to complex cellular and molecular controls with several ‘checkpoints’ from the blood to their destination in the brain parenchyma. As important components of the neurovascular unit, the functional state changes associated with lineage heterogeneity of myeloid cells are increasingly recognized as important for disease progression. In this review, we discuss some of the cellular elements associated with formation and function of the neurovascular unit, and present an update on the impact of myeloid cells on central nervous system (CNS) diseases in the laboratory and the clinic. We then discuss emerging strategies for harnessing the potential of site-directed myeloid cell homing to the CNS, and identify promising avenues for future research, with particular emphasis on the importance of untangling the functional heterogeneity within existing myeloid subsets.

## 1. Introduction

Diseases of the central nervous system (CNS) are some of the most devastating contributors to disease burden worldwide, and are associated with significant costs to healthcare and productivity [1,2,3]. Despite intensive research, treatment of pathology within the brain remains challenging due to its relative isolation from components of the circulatory system by the blood–brain barrier (BBB). Drug development for CNS disorders has been particularly challenging, as virtually all large molecules and the vast majority of small molecules identified in vitro cannot reach the parenchyma in vivo [4]. Accumulating evidence indicates that, rather than being completely separated from the peripheral immune system, the normal CNS undergoes tightly regulated surveillance by a variety of bone-marrow-derived immune cells, likely through several influx/efflux pathways [5,6,7]. This population of cells also appears to be crucial for neuronal development and function [8]. Also, the influx of leukocytes from the periphery is a hallmark of a number of autoimmune and neurodegenerative conditions, and despite sharing morphological characteristics of CNS-resident microglia, peripheral myeloid cells appear to possess non-redundant functions [6]. Importantly, in many cases the BBB is not simply “leaky”, as dyes and small proteins do not cross from the vasculature into the brain parenchyma [9]. This indicates that, under certain circumstances, leukocytes may in fact be invited guests to the CNS—participants in a coordinated series of interactions with the brain’s endothelial, glial, and epithelial barriers to contribute to brain homeostasis.

In many CNS diseases and in response to irradiation, the key cell type involved in trafficking to the parenchyma is the myeloid cell, a subset of leukocytes central to the innate immune system [10,11]. Myeloid cells are typically released from bone marrow as one of several subsets of monocytes, and following migration to target tissues may differentiate into macrophages or dendritic cells [12]. These cells possess a remarkable diversity of functions, which are dependent on their lineage, gene expression, surface molecules, and interactions with the surrounding environment via messenger molecules such as cytokines and chemokines secreted by other cells. Most notably, they are capable of destroying and clearing pathogens and debris independently via phagocytosis and secretion of proteolytic enzymes, and also of presenting antigens to the adaptive arm of the immune system [13]. This indicates that such cells could be primed for a function of interest via drug administration, genetic modification, or culture with certain molecules. Although transplantation into the CNS can be achieved directly via stereotactic injection, the damage induced by this process precludes it from routine use. Taking advantage of existing mechanisms of selective leukocyte extravasation into the brain parenchyma has emerged as a promising strategy for CNS cell and gene therapy, with tantalizing evidence of success in both preclinical and clinical applications.

The purpose of this review is to highlight the peculiar anatomy of the CNS and the unique mechanisms by which it allows myeloid cells to participate in site-directed homing, immune surveillance, and initiation of neuroprotective or neurotoxic activity. Additionally, promising developments in the use of myeloid-derived cells in the treatment of diseases of the CNS are discussed, and we outline some key targets for future research.

## 2. Contribution of Myeloid Cells to Central Nervous System (CNS) Homeostasis

### 2.1. The Resident CNS Myeloid Cell, the Microglia

Despite initial controversy as to their origin, it is now established that microglia are derived from early yolk sac erythromyeloid precursors and migrate to the brain during early embryogenesis [14,15,16], where they remain exclusively throughout life without substantial contribution from bone marrow-derived cells, unless recruited in response to distress signals from the CNS parenchyma and associated endothelial and glial barriers [11,17,18]. In their ramified state, microglia are far from ‘resting’, their processes continuously extend and retract to survey the surrounding parenchyma [19]. In their activated state, microglia- and CNS-infiltrating monocyte-derived macrophages both adapt an amoeboid morphology and share expression of many surface molecules including major histocompatibility class II (MCHII) complexes, which has made identifying the source of such cells in histological sections a challenge [10]. Recent data have established that mouse microglia possess a unique transcriptional and proteomic signature [20,21], including the unique expression of purinergic receptor P2Y12 [20,22] and transmembrane protein 119 [23] on the cell surface, now enabling specific labelling with antibodies. Evidence also indicates there are likely to be several heterogeneous subsets of microglia with region-specific functions, with a recent report highlighting a unique subset of fractalkine receptor (CX3CR1)-positive microglia present in the subventricular zone (SVZ), rostral migratory stream, and olfactory bulb that lacked ‘typical’ microglial markers Iba1, isolectin B4, and CD68, and appeared to be involved in the migration and integration of adult SVZ-derived neural progenitors into the olfactory bulb [24]. Other researchers have also used bone marrow chimeras and promoter-driven transgenic animals to label resident and infiltrating cells separately, and have found that in many conditions, the two subsets of cells vary considerably in their ability to phagocytose pathological deposits of protein, recruit other immune cells via antigen presentation, and contribute to pathology via production of neurotoxic substances [6].

### 2.2. Non-Microglial Myeloid Cells: Location and Transport in and out of the CNS

In the periphery, immune cells can traffic between lymph nodes and target organs via the blood and lymphatic vessels [25]. However, endothelial cells of CNS blood vessels are connected by tight junctions, which tightly control the passage of nutrients and cells across the vessel walls, and, with the exception of the meninges [26,27], the brain does not appear to have conventional lymphatic vessels, with entry and drainage of interstitial fluid (ISF) and cerebrospinal fluid (CSF) largely along narrow bulk flow channels in basement membranes of parenchymal blood vessels that do not support the passage of cells [28,29,30,31], specifically referred to as paravascular influx of CSF and intramural perivascular ISF drainage by Engelhart and colleagues in [7]. Also, the interfaces between the brain parenchyma, the vasculature, and the CSF-filled subarachnoid space are lined with a layer of astrocytic endfeet, which under normal conditions form an additional barrier to the transport of immune cells into the parenchyma [32].

**Figure 1:**
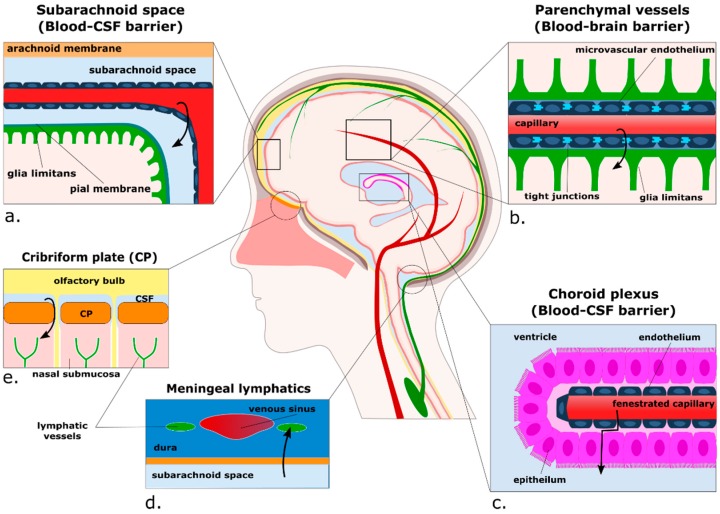
Putative routes for the extravasation and egress of myeloid cells from the brain parenchyma. Cells can traffic from blood to the subarachnoid space in the meninges (**a**), where they may traffic into the Virchow–Robin spaces at the surface of the brain. Secondly, cells can reach the brain parenchyma via small vessels such as capillaries (**b**) and post-capillary venules, where they must pass the tight junctions of the microvascular endothelium and breach the glia limitans. Thirdly, cells can reach the cerebrospinal fluid (CSF) through the choroid plexus (**c**). Cells carrying antigens towards lymph nodes have been proposed to follow at least two routes out of the brain compartment; into lymphatic vessels located in the meninges (**d**), and with CSF through the cribriform plate alongside olfactory nerves (**e**). Arrows represent the proposed direction of cell trafficking.

In the healthy CNS, non-microglial myeloid cells are found mostly within the subdural meninges, the choroid plexus, the CSF, and in the perivascular place between microvascular endothelial cells and the glia limitans of CNS blood vessels [28,33] (Figure 1). A recent fate-mapping study has uncovered that, contrary to previous belief, under normal conditions most macrophages in the perivascular and meningeal spaces derive from early fractalkine receptor (CX3CR1)-positive cells that migrate during embryogenesis and, like microglia, remain relatively stable once in their compartments [34]. The choroid plexus, by contrast, houses a population of shorter-lived cells that are gradually replaced in part by blood-borne monocytes. At this stage the functional relevance of these different subsets of macrophages is not known, but putative functions of non-parenchymal CNS myeloid cells as a whole include immune surveillance, regulation of CNS-specific T lymphocyte activity, and phagocytosis of molecules from the CNS parenchyma [5]. Interestingly, two recent studies using two-photon intravital microscopy have identified a small subset of cells residing in the juxtavascular parenchyma expressing either the classical dendritic cell marker CD11c or fractalkine receptor CX3CR1 with processes extended through the glia limitans into the perivascular space [35], or even further into the vascular lumen [36]. These reports extend on early identifications of microglia-like cells embedded in the glia limitans identified by electron microscopy [37]. Another proposed route for brain-to-peripheral communication pathway is the presentation of antigens to peripheral lymph nodes by myeloid cells migrating out of the CNS along with the CSF [7]. These routes include from the subarachnoid space through the cribriform plate into the lymphatics of the nasal submucosa [7,38], and the newly discovered meningeal lymphatic vessels, which are closely associated with venous sinuses in the dura mater and contain populations of immune cells in the steady state [26,27]. The relative contribution of these processes to presentation of antigen to peripheral lymph nodes has yet to be elucidated, but is likely to play an important role in the development of inflammatory disorders such as multiple sclerosis.

## 3. Passing the Checkpoints to the Parenchyma: A Focus on the Neurovascular Unit

During certain disease states and in response to radiation-induced disruption of homeostasis, circulating myeloid cells migrate into the brain parenchyma and surrounding spaces, where they may participate in phagocytosis and proteolytic digestion of macromolecules such as amyloid beta [39], or initiate the recruitment of effector cells such as T cells towards sites of pathology [40]. Their entrance to these compartments involves receptor-mediated chemotaxis and extravasation across the blood vessel endothelium, where they may (1) pass through the leptomeningeal vessels and epithelial cells of surrounding choroid plexus into the CSF-containing ventricles, which flow into the subarachnoid space; (2) enter the subarachnoid space directly via the leptomeningeal vessels; or (3) enter the perivascular space of arteries or post-capillary venules bordered by the glia limitans [41,42]. In the case of the latter, infiltrating cells participate in receptor-mediated chemotaxis towards astrocyte-released chemokines such as chemokine ligand 2 (CCL2), and release proteolytic enzymes to facilitate their passage through the basement membranes of the glia limitans [43,44]. The following section will discuss the unique anatomy of the perivascular niche and the sequential steps followed by leukocytes to pass the cellular and molecular checkpoints from the blood into the brain.

### 3.1. Anatomy of the Perivascular Niche

**Figure 2:**
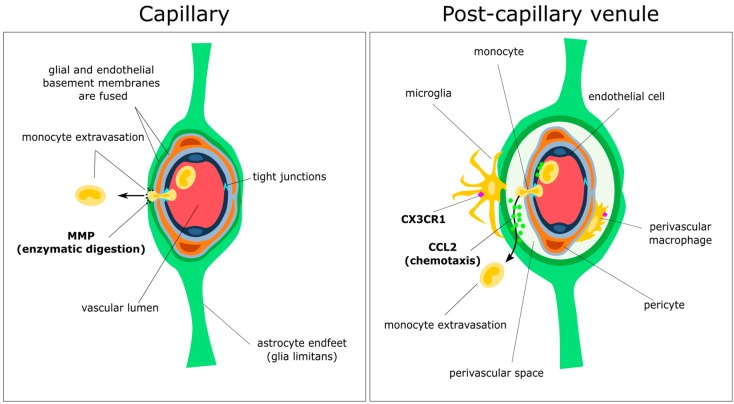
Simplified schematic drawing of the anatomy and function of the perivascular niche. The blood–brain barrier consists of a layer of microvascular endothelial cells connected by selective tight-junctions that prevent the passage of most large molecules and cells. These junctions rapidly remodel to allow passage of leukocytes such as monocytes through to the glial and endothelial basement membranes. An important distinguishing feature between capillaries and post-capillary venules is the separation of glial and endothelial basement membranes in the latter (right), forming the perivascular space. Also embedded in the endothelial basement membrane are pericytes, a population of mesenchymal cells responsible for maintaining endothelial cell formation and function, and polarising astrocyte endfeet. Perivascular macrophages (labelled, right) may derive from embryonic precursors, or may arise from infiltrating monocytes that have passed through the post-capillary endothelium. To reach the CNS parenchyma, cells must pass through the glia limitans, consisting of astrocyte endfeet attached to a basement membrane (arrows). This is achieved in part by coordinated chemotaxis with chemokines such as chemokine ligand 2 (CCL2; right arrow), and enzymatic digestion of intact basement membrane by matrix metalloproteinases (MMPs; left arrow). Also pictured are rare fractalkine receptor (CX3CR1)-positive parenchymal cells that appear to extend processes through the glia limitans, possibly to sample antigen from the perivascular niche.

The perivascular niche is a major site of influx of leukocytes during the induction of experimental autoimmune encephalitis (EAE) [7,40], and is an important site of amyloid-beta deposition in Alzheimer’s disease (AD) and cerebral amyloid angiopathy (CAA) [30,45], indicating this region is a key target for resolution of brain pathology. The perivascular niche is a multi-layered structure composed of numerous interdependent cell types, which varies according to vessel type and location [7]. The post-capillary venules are the main site of leukocyte extravasation into the inflamed CNS and are the primary site of “inflammatory cuffs” observed in models of EAE [40] (Figure 2). Nearby capillaries, by contrast, do not have a “perivascular space” as the basement membranes of endothelial and glial barriers are fused [7].

Enclosing the lumen of the post-capillary venule is a layer of specialised microvascular endothelial cells attached to a basement membrane connected by tight junctions intermingled with adherens junctions, which serve to regulate paracellular diffusion of ions and maintain cell–cell contacts, respectively [46]. The distinct morphology of the endothelial cells and associated zipper-like junctions serve to tightly regulate the movement of nutrients and cells by forming an extremely selective physicochemical barrier [46,47]. The tight junctions themselves are composed of a network of transmembrane proteins including occludin, claudins, and junctional adhesion molecules [47]. These proteins are organized and linked to the cortical actin cytoskeleton by intracellular scaffolding proteins such as zonula occludens-1. For a review of tight and adherens junction components and their roles in maintenance of the BBB, see [46]. Embedded in the endothelial basement membrane are pericytes, thought to be derived from neural crest cells in the neuroectoderm, and mesenchymal stem cells in the mesoderm [48]. Pericytes are identified based on the expression of a number of molecules including platelet-derived growth factor receptor-β (PDGFR-β) [49], regulator of g-protein signalling 5 (RGS5) [50], and Aminopeptidase N (CD13) [51,52]. More reliably, pericytes can be identified by their ultrastructural localization to the endothelial basement membrane in semi-thin electron microscopy sections [53]. Although a number of studies have suggested that cells expressing typical pericyte markers can migrate and differentiate into antigen-presenting cells in response to ischemia or brain injury [54,55,56,57], there is still the possibility that experimental conditions as such may induce the expression of the aforementioned ‘pericyte’ markers on myeloid cells [53]. Thus uncertainty remains over their lineage and differentiation capacity [55,58]. Of the many roles ascribed to pericytes, there is strong evidence for a crucial role in the formation and function of the BBB, participating in structural support of the mirovasculature, vasodynamic control, basement membrane synthesis and repair, organization of endothelial cells, and the polarization of astrocyte endfeet associated with the glia limitans [48,59,60]. Inside the perivascular space are a population of fractalkine receptor-positive perivascular macrophages derived from early embryonic progenitors [34]. Under certain circumstances, particularly following irradiation or injury, these cells may also be replaced by blood-borne progenitors [61,62,63,64,65]. Enveloping the perivascular space is the glia limitans, a barrier formed by astrocyte endfeet and surrounding basement membrane. This barrier is the final checkpoint for infiltrating immune cells to enter the brain parenchyma, and is a crucial regulator of inflammatory processes and dysfunction of nearby neural tissue. For a review of molecular mediators of astrocyte barrier function, see [66].

### 3.2. Initial Attraction: Monocyte–Endothelium Interactions

The migration of peripheral immune cells through the cerebral microvasculature follows the same general course as in the periphery, starting with selectin and adhesion molecule-mediated tethering and rolling of leukocytes to the endothelium, where they are activated, encounter integrins and immobilized chemokine ligands, and, if recognized by their cognate receptors, will tightly adhere to the endothelium and extend processes through the intercellular junctions to initiate diapedesis [39,67]. Although this remains to be confirmed in vivo, evidence from in vitro experiments indicates that leukocytes may extravasate across the BBB endothelium in a paracellular (between cells) or transcellular (through cells) fashion, with paracellular transport more common for monocytes and T cells, and an apparent preference for tri-cellular junctions over the bi-cellular route [68,69]. By contrast, neutrophils appear to favour the transcellular route through simulated blood-brain and blood-CSF barriers [70,71]. Diapedesis, the passage of cells through an intact endothelial barrier, is associated with rapid remodelling of tight junctions, such that as the leukocyte travels through the junction, the gaps are rapidly replaced with membrane from the endothelial lateral border recycling compartment, and the leukocyte itself forms transient connections with unligated cell adhesion molecules such as PECAM1-1 and CD99 [68]. This serves to preserve BBB integrity and prevents uncontrolled influx of solutes, proteins and other cells from the periphery during the process of extravasation. Thus the leukocyte has passed the first ‘checkpoint’ through to the perivascular space without significantly disrupting homeostasis.

### 3.3. Gatekeepers: Astrocytes and the Glia Limitans

Once inside the perivascular space, infiltrating immune cells may either remain within the perivascular space, or may go on to breach the glia limitans. This is particularly pertinent in the case of EAE, where activated T lymphocytes and monocyte-derived macrophages are known to breach the glia limitans of the spinal cord through proteolytic cleavage of dystroglycan, laminin, extracellular matrix, and other membrane proteins by matrix metalloproteinases (MMP-) 2, 7, and 9 [44]. Astrocytes also release MMP-7, though to a lesser extent than myeloid cells [72]. Nonetheless, this may indicate that the glia limitans itself is “inviting” the entry of immune cells from the perivascular space.

### 3.4. Spotlight on Coordinated Chemotaxis: The CCL2/CCR2 Pathway

This concept of “invited” migration to the brain parenchyma during demyelination is even better demonstrated with new insights gained regarding chemokine ligand 2 (CCL2), a crucial chemokine for egress of monocytes from bone marrow, and their transport through both endothelial and glial checkpoints to the brain parenchyma. Although many chemokines are expressed in the neurovascular unit in response to pathological insults, CCL2 has received considerable attention as the ligand and its receptor are essential for migration of the largest populations of leukocytes across the BBB in many mouse models of injury and disease [43,73,74,75,76,77,78], and expression of CCL2 in response to brain pathology is associated with production of many associated metalloproteinases and cytokines such as MMP-10, MMP-12, IL-1, and TNF-α [78]. Many cells in the neurovascular unit can release CCL2 in response to insult or injury. These include neurons [79,80], astrocytes [43,73], microglia [73,81], and microvascular endothelial cells [43]. The coordinated expression of chemokines from these cells guides the extravasation of leukocytes from the vasculature, and towards the site of action. For example, injured neurons in the superficial cervical ganglia express *Ccl2* mRNA, but the ligand itself is found localized to nearby microvascular endothelial cells, in which the mRNA expression of *Ccl2* is undetectable [79]. This appears to indicate that CCL2 has been released by the neurons and has travelled through the extracellular space to the endothelial cells, where they bind to receptors and are stabilized for presentation to infiltrating leukocytes. Another elegant study illustrates the relative contribution of CCL2 release from astrocytes and endothelial cells in the recruitment of leukocytes in EAE, by comparing astrocyte and endothelial-specific *Ccl2*-knockout mice [43]. When astrocyte CCL2 is absent, infiltrating cells become trapped in the perivascular space, reducing the severity of the pathology but maintaining the same time of onset. Conversely, if endothelial cell CCL2 is absent, the pathology is delayed but similar in severity, with an apparent stalling of leukocytes in the vascular lumen. In this case it is possible that the smaller chemokine gradients induced by the comparatively distant release of CCL2 by astrocytes (and possibly other cells) was able to preserve leukocyte influx to the parenchyma, albeit less efficiently. In summary, both major checkpoints of the neurovascular unit appear to participate in the coordinated influx of immune cells from the periphery. Whether this is a beneficial or detrimental response depends on the specific pathology, thus, chemokine-mediated leukocyte influx remains a complex, yet promising target for therapeutic intervention.

## 4. Myeloid Cells: The White Knights of CNS Therapy?

The field of neuro-immunology has undergone a major shift in recent decades, sparked by the recognition that infiltrating leukocytes and resident microglia can have both harmful and trophic effects on diseased neural tissue. Immunotherapies are gaining traction as promising strategies for treating a wide variety of diseases, with many clinical trials underway. However, therapeutic strategies are yet to mature, likely because the true complexity of immune dysfunction within the CNS during disease is not fully understood. In this section we will discuss the progress of research targeting the transplantation of the myeloid family of cells for the resolution of a selection of diseases affecting the CNS.

### 4.1. Genetic Deficiencies: Lysosomal/Peroxisomal Storage Diseases

Lysosomal storage diseases (LSDs) and peroxisomal storage diseases (PSDs) are rare, typically fatal, multisystem childhood disorders usually resulting from a genetic deficiency of one or more lysosomal/peroxisomal enzymes [82,83,84]. A major component of many LSDs/PSDs is progressive neurodegeneration associated with accumulation of macromolecular substrates and associated neuroimmune activity [85]. Bone marrow transplantation has been used for decades as a treatment for MLD/LSDs; however, outcomes remain suboptimal, with incomplete resolution of symptoms, graft-versus-host disease, and death still commonly reported [86]. In animal models, it is the F4/80+ myeloid cells that migrate into the brain parenchyma and phagocytose aberrant deposits of lipid and other substrates following bone marrow transplantation, indicating that they are likely to be the driver of therapeutic action [83,87,88,89,90]. Evidence from these experiments indicates it may be possible to enhance treatment to prevent the impairments in motor conduction associated with LSDs by transplanting gene-modified hematopoietic stem cells (HSCs) over-expressing the missing gene. Other experiments have shown that this effect is enhanced when gene expression is tied to the CD11b (myeloid-specific) promoter [91], in this case correcting both proteoglycan build-up and open-field mouse behaviour.

Furthermore, the first clinical trial of autologous HSC transplantation with lentivirally transduced, “supertherapeutic” *ARSA* gene expression in pre-symptomatic children with arylsulfatase A (ARSA) deficiency (the cause of metachromatic leukodystrophy) was recently completed, and appears to have been successful at preventing the demyelination associated with sulfatide accumulation for at least two years post-therapy [92,93]. This promising data indicates myeloid cells are likely to be a key target for gene therapy; however, longer term follow-up and larger studies will be needed to determine whether the strategy successfully prevents the progression of disease. Also of interest would be whether this strategy would be capable of arresting disease progression in patients already affected by the disease.

### 4.2. Neurodegenerative Diseases

Many neurodegenerative diseases are characterized by the aggregation of proteins and peptide fragments within the brain, and the impaired clearance of these products is hypothesized to underlie the pathogenesis of these diseases [53], although their role as initiators of disease remains controversial [94,95,96]. Several pharmaceutical companies are progressing through clinical trials utilizing targeted immunotherapy against aggregated protein products, either through vaccination or antibody administration, with limited evidence of success. Initial data suggests these therapies are unlikely to be a ‘magic bullet’ for neurodegeneration, as dramatic clinical improvement has yet to be shown in phase III trials, and side effects have been relatively common [97,98]. In this section we discuss the rationale and preclinical evidence behind targeted myeloid-based cell therapy for a selection of neurodegenerative disorders, which may have the potential to enhance the clearance of protein and peptide fragments from the CNS.

#### 4.2.1. Alzheimer’s Disease

Alzheimer’s disease (AD) is the most common form of dementia worldwide [99]. The hallmarks of AD are progressive loss of neurons and synapses, associated with the presence of amyloid beta (Aβ) plaques throughout the brain parenchyma and around blood vessels, and tau neurofibrillary tangles [100]. In common with other brain pathologies, models of AD are associated with the presence dystrophic microglia with characteristics of an activated state, such as an amoeboid morphology and expression of MHC antigens, but with unique hallmarks of dysfunction including ultrastructural signs of oxidative stress [101,102]. With the suggestion that dystrophic microglia accumulate over the course of ageing and in many cases of neurodegenerative disease, microglial age-related senescence was proposed as a key contributor to neurodegeneration. Microglial dysfunction appears to precede alterations in processing and subsequent aggregation of Aβ, and has even been suggested to underlie the disease itself [102,103,104]. In line with this theory, activation of complement cascade and aberrant pruning of synapses by dysfunctional microglia are early events preceding the appearance of overt pathology in some animal models of familial AD [105], and apparently occurs independently of neuronal protein aggregation [106]. Several animal models of familial AD have nonetheless shown that infiltrating monocyte-derived macrophages may be intimately involved in restricting disease progression, possibly via the phagocytic clearance of Aβ [107,108,109]. Despite the controversy, clearance of aggregated proteins remains a promising strategy for disease modification.

It has become evident that resident microglia and blood-derived macrophages behave differently in the presence of Aβ [45]. Although microglia appear to internalize as much Aβ as peripheral macrophages in vitro, subsequent lysosomal fragmentation is slow, incomplete, and easily overwhelmed by the presence of large amounts of protein [110]. Thus it is possible that infiltration of peripheral myeloid cells is a protective response to amyloid loading. Interestingly, common genetic risk factors indicate that impaired clearance of Aβ by macrophages may be a central part of disease pathogenesis. For example, rare mutations in the *triggering receptor expressed on macrophages-2* (*TREM2*) gene are associated with an increased risk of developing neurodegenerative diseases including AD, and many mouse models of familial Alzheimer’s display alterations in *Trem2* expression [111,112]. Although animal models report seemingly conflicting effects of eliminating or overexpressing *Trem2* [113,114], and the relative contribution of peripheral and CNS-resident cells is yet to be investigated, most studies agree that the receptor (and by extension, myeloid cells) plays an important role in the response to deposition of Aβ in AD, likely though effects on cell surface transport and phagocytosis [115,116]. The most extensively recognized gene locus for AD risk is the *APOE* gene, with the *APOE4* allele considered risk-inducing, and *APOE2* generally considered protective [117]. Macrophages are known producers of apolipoprotein, and in vitro challenge of macrophages from AD transgenic mice expressing the three major isoforms of *APOE* indicates that the *APOE2* allele may confer an enhanced ability to phagocytose Aβ on myeloid cells associated with increased expression of MMP-9 [118]. Additionally, transplantation of *APOE3*, but not *APOE4* expressing bone marrow improves pathology and behavioural changes in double-transgenic AD mice in vivo [119]. Another strategy used in vivo was to disrupt transforming growth factor-beta (TGF-β)-mediated transcription regulation driven by the myeloid-specific CD11c promoter, which resulted in major attenuation of plaque formation (~90%) and improvement of behavioural deficits in Tg2756 AD mice [108]. This was associated with increased infiltration of macrophages around cerebral vessels and amyloid plaques in vivo, and enhanced phagocytosis of Aβ by macrophages in vitro. Overall it appears that enhancing the Aβ clearance capacity of macrophages by increasing lipid binding or proteolytic enzymes, or perhaps replacing senescent microglia with enhanced cells to avoid unwanted bystander damage, might become a strategy for CNS tissue regeneration.

Although current therapeutics approved for use in mild to moderate AD purportedly target neurons directly, several lines of evidence indicate the myeloid lineage of cells may be additional targets of these drugs. For example, acetylcholinesterase inhibitors have been used to enhance cholinergic neurotransmission, and have been modestly successful at delaying cognitive decline [120]. Both blood-borne macrophages and resident microglia possess alpha 7 nicotinic acetylcholinergic receptors, and their activation on microglial cells prevents Aβ-induced oxidative stress and facilitates clearance of Aβ in vitro [121,122]. Thus it is tempting to speculate that the immunomodulatory properties of these drugs may be contributing to their modest success. At present, a popular strategy for eliminating Aβ in clinical trials is targeted immunization involving either an active vaccine against specific Aβ fragments, or intravenous antibody infusion. The first vaccination trials with AN1792 were discontinued due to the induction of meningoencephalitis associated with T-cell infiltration in several patients [123], and treatment only elicited a sufficient antibody response in ~50% of patients [124]. Additionally, cerebral amyloid angiopathy persisted despite clearance of cortical plaques in more than one case [125,126]. Similar rates of response have been observed in trials of a newer vaccine designed to avoid the T-cell specific response observed in the AN1792 trial [127]. Given the limited response rates to active vaccination (potentially due to age-related immune senescence), direct administration of antibodies has been tested, but none of the phase III trials so far have demonstrated significant improvements in symptoms or slowing of cognitive decline [97,98]. Interestingly, both animal models and clinical trials have also demonstrated that CAA can persist after monoclonal antibody-mediated parenchymal clearance of Aβ [128,129]. Although this is likely to depend on the specific target peptide fragments, it does indicate that stimulating an endogenous immune response may not be sufficient to arrest the progression of vascular Aβ deposition once it has already progressed, and perhaps that the neurovascular unit may be a particularly important target for therapy in the future.

Although no transplantation trials appear to be in progress, one phase II trial is currently underway investigating use of Granulocyte-Macrophage Colony-Stimulating Factor (Sargramostim; GM-CSF) for the mobilization of hematopoietic progenitors in patients with AD (NCT01409915). Animal trials have indicated that improvements in amyloid load and cognitive function following this treatment is associated with increased density of Iba-1 positive cells around plaques [130]. Although the authors did not distinguish resident microglia from infiltrating myeloid cells, which also express Iba1, it is possible that increased influx or priming of peripheral myeloid cells is driving this response. Indeed, in vitro evidence suggests GM-CSF treatment of monocytes enhances their capacity to traffic across endothelial barriers resembling the BBB, and promotes the release of reactive oxygen species and interleukin-6 [131].

In summary, although the precise roles of specific myeloid subsets in the promotion or response to neurodegeneration are yet to be fully untangled, accumulating evidence indicates that myeloid cells from both within and outside the CNS have important roles to play in the modulation of neurodegenerative processes. Thus, the myeloid lineage remains a key target for therapeutic strategies, whether used to replace or support senescent microglia, or to facilitate clearance of aggregated proteins.

#### 4.2.2. Amyotrophic Lateral Sclerosis (ALS)

ALS is a neurodegenerative disease characterized by the progressive loss of motor neurons in the cerebral cortex, brain and spinal cord, resulting in paralysis and eventual death [132,133]. Many gene mutations have been identified in familial cases, which has enabled the generation of transgenic models to investigate pathogenesis and treatment options [134]. Evidence from *superoxide dismutase-1* (*SOD1*) mutant mice suggests that non-neuronal cells contribute substantially to the neurodegenerative process, and that myeloid cells in particular play an important role [135,136]. Although there appears to be some disagreement over the role of monocyte infiltration in ALS progression, it appears the phenotype of immune cells interacting with motor neurons changes throughout the course of disease, indicating they could be a useful target for therapy [132,137]. Initial evidence for this line of enquiry has come from a recent study, which has identified major alterations in composition and function of peripheral monocytes in the blood of 10 ALS patients compared to controls [138]. Researchers found blood from patients displayed an increase of classical, “inflammatory” CD14++CD16− monocytes relative to non-classical CD14+CD16++ cells. In vitro, the CD16− monocytes from these patients were significantly less efficient at phagocytosis of yeast-derived particles, and more inclined to adhere to collagen-coated chamber walls under shear stress. Post-mortem investigations also indicated an increase in migration of monocyte derived cells into the spinal cord of ALS patients. This raises the possibility that in ALS, neurodegeneration is accompanied by an inefficient innate immune response, which could be modulated by selective targeting of myeloid cells.

Clinical success for ALS has been limited. The drug most commonly used to address ALS, Riluzole, appears to prolong patient survival by several months on average, with only small improvements in motor function [139]. Riluzole is purported to reduce excitotoxicity associated with ALS via the inhibition of neuronal glutamate release, in part through its actions on voltage-dependent sodium channels [140]. Interestingly, a recent study has demonstrated that in vitro, Riluzole is capable of modulating responses of rat microglia to inflammatory stimuli, possibly via activation of two calcium-activated potassium channels, SK3 (KCa2.3) and SK4 (KCa3.1) [141]. Specifically, administration of the drug attenuated “pro-inflammatory” cytokine mRNA expression in response to lipopolysaccharide challenge, and enhanced expression of *brain-derived neurotrophic factor (BDNF*) mRNA following stimulation with interleukin-4. Although further study is required to identify the sites of action and therapeutic relevance of this effect, this study has highlighted a previously under-recognized molecular pathway for neuroprotection.

Although therapeutic strategies targeting the myeloid lineage are yet to gain significant attention in the clinic, several lines of evidence suggest it may gain traction in the future. For example, *SOD1* mutant mice transplanted with bone marrow stimulated by granulocyte colony stimulating factor (G-CSF) were partially protected from motor neuron loss in the ventral horn of the spinal cord [142]. This was accompanied by an influx of myeloid cells with a “microglial” phenotype, and increased expression of neurotrophic factors such as glial cell line-derived neurotrophic factor (GDNF), brain-derived neurotrophic factor (BDNF), vascular endothelial growth factor (VEGF), and angiogenin. Similar results were obtained in double-transgenic, immune cell-deficient mSOD1^G93A^/PU.1^−/−^ mice, in which transplantation with wild-type bone marrow was associated with a slowing of disease progression compared to controls transplanted with marrow from mSOD1^G93A^ mice [136]. Also, a phase 1 trial using NP001 (sodium chlorite) to downregulate inflammatory pathways in myeloid cells was conducted recently based on promising preclinical results, with preliminary results suggesting it may slow disease progression [143]. If successful, this approach underscores the potential for myeloid cell-based therapies for ALS.

### 4.3. Autoimmune Diseases: Multiple Sclerosis

Multiple sclerosis (MS) is a heterogeneous, chronic demyelinating disease affecting the CNS, with unknown aetiology [144]. MS pathogenesis is characterized by early infiltration of leukocytes into the brain and spinal cord, leading to discrete lesions and loss of associated axons [47]. Although T cells play a central role in the progression of MS and its classical animal model experimental autoimmune encephalomyelitis (EAE), innate immune activity is critical for the progression and resolution of demyelination [145]. This involves marked but temporary monocyte extravasation across CNS gateways to the parenchyma [146], and secretion of cytotoxic factors such as inflammatory cytokines and reactive oxygen/nitrogen species by infiltrating inflammatory monocyte-derived macrophages during the initiation of demyelination (the effector phase) [147,148]. Factors important for the remyelination process include microglia/macrophage phagocytosis of debris and stimulation of oligodendrocyte progenitor cell differentiation, via clearance of axonal debris and release of neurotrophic molecules such as Activin A [147,149,150]. Also of note is the role of antigen presentation to primed T cells by CD11c+ (primarily dendritic) cells located at the meninges and perivascular spaces during the effector phase of EAE [151,152]. Historically, the distinction between resident and infiltrating myeloid cells has been complicated by overlapping phenotypic characteristics of reactive microglia and infiltrating monocyte-derived macrophages, as well as confounding effects associated with conditioning for transplantation of labelled blood-derived cells [153]. Recently, a study employing targeted expression of CCR2/CX3CR1 fluorescent reporters has uncovered distinct patterns of behaviour and associated gene expression of CCR2+ monocyte-derived macrophages and resident CX3CR1+ microglia during the induction phase of EAE [154]. Blood-derived cells were found in direct contact with nodes of Ranvier and adopted a distinctly pro-inflammatory gene expression profile, whereas microglia underwent marked downregulation of metabolic genes, made direct contact with macrophages but not with axons, and appeared to be taking up myelin debris left by the infiltrating cells. This raises the possibility that microglia may be particularly suited towards neuroprotection during the initiation of MS, and indicates their gene expression profile may uncover some targets that could be modified in blood-derived cells for therapeutic purposes. At this stage the contributions of myeloid subsets to the resolution of demyelination are less clear, although both resident and infiltrating cells appear to play a role in oligodendrocyte differentiation, and evidence points towards ‘M2’ polarization as beneficial [153]. The heterogeneous phenotypes of macrophages along the ‘M1-M2’ spectrum might be one of the reasons for such contradictory results in animal models of MS [155]. This complexity is reflected in a study of tissue from multiple sclerosis patients, in which a significant number of myeloid cells within chronic lesions expressed markers typical of both M1 and M2 states, namely CD40 and mannose receptor [156]. It could be argued that these descriptors of polarization states as M1/M2, or pro/anti-inflammatory are insufficient to properly characterize the parenchymal myeloid cells response to stimuli [157]. It is also possible that this heterogeneity reflects the developmental specialization of specific myeloid subsets, which has not yet been fully characterized.

Currently, the α4 integrin antibody natalizumab is used for management of relapsing-remitting and progressive multiple sclerosis, with its therapeutic mode of action purported to involve its inhibitory effect on T cell adhesion to vascular endothelium and subsequent extravasation into the brain parenchyma [158,159]. Monocytes also employ α4 integrins to cross the blood–brain barrier [160], indicating they would be affected in a similar manner to therapy. Unfortunately, natalizumab therapy carries a risk of potentially fatal progressive multifocal leukoencephalopathy caused by the reactivation of latent John Cunningham (JC) virus, making it unsuitable for patients with latent infection [161]. Interferon beta, an important immunomodulatory cytokine, has also been used to prevent or reduce the number of MS relapses [162]. Although its many mechanisms of action are not fully understood [163], in a rat model of EAE, interferon beta treatment was associated with a marked reduction of monocyte infiltration to the perivascular space and concurrent downregulation of adhesion molecule expression [164]. Studies have also shown that interferon beta can downregulate antigen presentation in cultured peripheral and CNS-resident APCs via reductions in MCHII expression and alterations in cytokine signalling [165], indicating its therapeutic activity may occur in the meninges, choroid plexus and perivascular spaces, which are crucial locations for antigen presentation to T lymphocytes in EAE [166]. Additionally, interferon beta appears to upregulate microglial clearance of myelin and axonal debris [167,168]. Although moderately successful, therapy with interferons is associated with numerous side effects, including a significantly increased risk of depression and suicidal behaviour [162,169]. This is likely due to the broad range of effects of supraphysiological systemic doses of an endogenous cytokine.

A number of clinical trials are currently underway utilizing the existing capabilities of immune cells to modulate immune response in MS. Firstly, a trial of autologous hematopoietic stem cell transplantation after non-myeloablative chemotherapy was recently conducted [170], with the aim to eliminate autoreactive T cells and re-establish self-tolerance. Early reports indicate improvements in symptoms and reduced frequency of relapses; however, this will need to be followed longer term to determine whether the effect persists well beyond the usual course of disease. Particularly of interest is whether ‘resetting’ the patient’s immune systems in this way is sufficient to halt reactivation of T lymphocytes, or whether the transplanted cells may require enhancement to resist influence from dysfunctional signalling outside of the hematopoietic compartment. A more targeted therapeutic approach currently under investigation involves the use of tolerogenic dendritic cells (tolDCs) for the suppression of cytotoxic T cell responses [171]. Although dendritic cells have long been recognized as potent antigen-presenting cells, it has only recently been discovered that they can also regulate autoreactive T cells via the induction of apoptosis, or by modulating their phenotype towards a T-regulatory (Treg) cell profile [172]. Despite challenges with inducing a stable tolerogenic phenotype in vitro, one laboratory has developed a method of inducing human and mouse tolDCs [173,174], and has successfully applied MOG-loaded tolDCs to a mouse model of EAE with promising results [174,175]. Further research, in particular the upcoming phase I clinical trial (NCT02618902), is anticipated with interest.

### 4.4. Brain Tumours: Glioma

Malignant gliomas are aggressive tumours arising from cells of astrocyte, oligodendrocyte or ependymal origin, comprising the most common form of malignant tumours on the CNS [176]. Prognosis is poor and recurrence is common, with chemotherapy, radiation, and surgical resection only modestly extending average survival times [177,178]. Gliomas evade detection by inducing robust local and systemic immunosuppression, and secreting immunosuppressive chemokines, prostaglandins, and other ligands to protect themselves against adaptive and innate immune responses [179]. This is associated with increases in circulating T regulatory cells (Tregs) [180] and myeloid-derived suppressor cells (MDSCs) [181], which are recruited to tumours by chemokines such as CCL2 released by tumour-associated microglia and macrophages [182]. Tumour-associated myeloid cells contribute significantly to the tumour mass, and have been proposed to promote tumour growth, survival, and invasion of surrounding tissues by producing growth factors, cytokines, and proteolytic enzymes [179,183]. Alkylating agents such as temozolomide (TMZ) are the standard therapy for glioblastoma, and commonly result in profound myelosuppression [184].

Although myeloid cells undergo a distinct phenotypic shift on contact with the tumour microenvironment, evidence suggests their activity may be required for protection against tumour progression. A number of studies have demonstrated beneficial effects of macrophage/microglial activation strategies such as Stat3 inhibitors [185] and Amphotericin B on protective responses to glioma. Another study demonstrated that ganciclovir-mediated reduction in CD11b+ macrophages led to increases in tumour volume in a syngeneic model of glioma, associated with reductions in TNF-expressing cells and lymphocytes [186]. It could be speculated that incomplete removal of myeloid cells in this study has spared particular subsets with especially detrimental effects on glioma in isolation. In support of this, two recent studies have investigated the role of brain resident CD11b+ cells in the contribution to glioma progression, and have shown that resident rather than infiltrating myeloid cells are the primary contributors to tumour growth and angiogenesis. In these studies, head-shielded irradiation with or without selective ablation of CD11b+ microglia via intraventricular ganciclovir infusion enabled the examination of relative contribution of resident and infiltrating myeloid cells in GFP+ bone marrow chimeras [187,188]. Brain-resident cells comprised the majority of tumour-associated macrophages, and their selective reduction slowed tumour growth and decreased blood vessel density. This suggests microglia may be particularly responsible for the progression of glioma, and raises the possibility that infiltrating myeloid cells are in fact protective. To confirm this hypothesis, further phenotypic characterization will be required to identify cells as microglia. Once the phenotypic and functional diversity of glioma-associated myeloid cells has been clarified, it may be possible to target specific subsets directly to enhance protective functions or mitigate dysfunctional interactions with the tumour microenvironment.

One strategy for overcoming robust immunosuppression in the glioma microenvironment is enhancing the presentation of tumour-associated antigen to T cells prior to encountering the tumour. In humans, this strategy has mostly revolved around dendritic cell-based vaccines, which were initiated for other cancers in the 1990s and have undergone various modifications to improve immunogenicity [189]. Currently there are dozens of phase I–II clinical trials employing vaccination with autologous dendritic cells loaded with whole tumour lysate, and purified or synthetic antigen peptides [190,191,192,193], with some indications of success associated with decreases in number of Treg cells following treatment [194]. However, as of yet none have been particularly successful at addressing the poor antigen-specific T cell response rates observed so far. This is possibly due to systemic immunosuppression caused by the glioblastoma itself [195]. In the future it may become possible to enhance dendritic cell function by altering genes involved in antigen presentation or response to systemic immunosuppression, or it may be necessary to target a different subset of cells for optimal response, such as tumour-associated macrophages/microglia [196]. Interestingly, transplantation of gene-modified hematopoietic stem cells (the precursors to infiltrating macrophages) has also been tested in humans, but only for the purpose of mediating the myeloablative side-effects of high dose chemotherapy [197,198]. Nonetheless, these studies demonstrate that HSC transplantation is feasible, which indicates that gene therapy involving HSCs may be a promising avenue for further investigation.

## 5. From Bench to Bedside: Targets for Future Research

Since the role of immune surveillance in the brain was first recognised, much has been learned about the role of myeloid cells in the initiation and resolution of CNS disorders. In particular, the past several years have seen the development of new, reliable methods of genetic modification that have greatly accelerated our understanding of the CNS during health and disease. Despite this, current strategies for harnessing the potential of the myeloid cell are yet to mature into reliable, efficacious treatments.

We suspect this is because we are only just beginning to understand the true heterogeneity of the hematopoietic system, particularly relating to cells found in the neurovascular unit. Despite recent insights regarding the ontogeny and phenotype of these cells, many researchers continue to refer to all phagocytic cells in the CNS as “microglia”. Therefore readers are strongly encouraged to evaluate the literature regarding neuro-inflammation and microglial activation with care, and to refrain from equating microglia and CNS macrophages in their own research. This can now be achieved relatively easily, as many reporter systems are available [10], and the transcriptional profiles of the two major subsets are well characterized [22,23,24,34]. Additionally, novel methods of depleting specific subsets of cells are under development [199], which may assist with the functional characterization of the heterogeneous subsets of microglia and other myeloid cells without confounding effects of irradiation on brain tissue, for example [200]. Additionally, a closer examination of the heterogeneous states between the two poles of M1 and M2 activation in macrophages is needed to understand which factors are most desirable for neuroprotection. M2 activation is generally considered the desirable, neurotrophic phenotype, and M1 macrophages are thought to encourage neurodegeneration. However, growing evidence suggests that both phenotypes, or perhaps an intermediary state, are required for different phases of the neuro-immune response [201]. In addition, this schema may be particularly inappropriate for characterizing responses of resident microglia to pathological insults [157]. Undoubtedly, progress in fate mapping and identification of the various subsets of myeloid cells will enable us to explore this issue further.

As cell-based therapies enter the clinic, new strategies for monitoring transplanted cells in vivo will be required. The capacity to monitor the spatial distribution of myeloid cell homing will enable clinicians to monitor the co-localization of transplanted cells with existing biomarkers of pathology, which may enable them to examine outcomes earlier than is currently possible. The development of new imaging modalities and reporter systems has progressed far in animal trials; however, challenges associated with retention of labels, sensitivity, long-term safety, and viability of reporters have limited its use in the clinic to date [202]. Until this has been optimized, clinicians may benefit from the use of existing biomarkers of neuro-immune activity such as translocator protein (18 kDa, TSPO) expression for the localization of activated microglia and infiltrating myeloid cells during disease and therapy [203]. Positron emission tomography using TSPO ligands as markers of microglial activation have been investigated for use in neurodegenerative diseases and multiple sclerosis [204,205], and are under investigation for prediction and monitoring of disease progression [203,206]. Molecular imaging with TSPO ligands has also been applied for non-invasive monitoring of glioma in a null-background mouse model [207], and is under consideration for monitoring of key events in glioma progression in humans [208]. Future work should focus on novel applications for existing non-invasive imaging techniques, or identify additional strategies for non-invasive monitoring of cell trafficking and associated neuro-immune activity.

**Figure 3:**
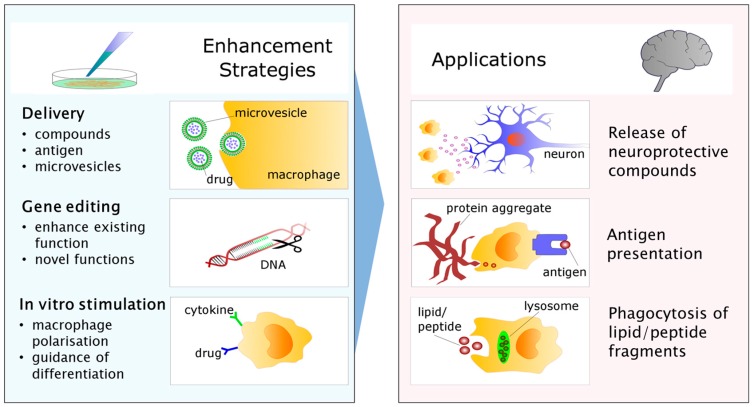
Strategies for enhancing myeloid cell therapy for the CNS. The phagocytic capacity of myeloid cells may be adapted for use as delivery vehicles for compounds, antigens, or microvesicles loaded with therapeutic substances destined for the brain. Pictured is a macrophage in the process of taking up drug-loaded nanoparticles intended for delivery into the CNS. Gene editing enables the up/downregulation of existing functions to enhance protective, or eliminate damaging responses to disease. It is also possible to introduce expression of enzymes not usually present in these cells through this process. Another strategy for guiding myeloid cell-based therapies in the CNS involves in vitro manipulation of polarisation or differentiation, for example with cytokines or drugs known to guide the cells into a more neuroprotective phenotype. As a result of in vitro manipulation, myeloid cells returned to the circulation may then be better capable of ameliorating brain pathology by one of several mechanisms. For example, CNS-infiltrating myeloid cells may be better equipped to release compounds with growth or survival-promoting effects on nearby diseased neurons. Alternatively, it may be possible to enhance the capacity of infiltrating cells to present brain-derived antigens to the adaptive immune system, thus enhancing clearance of unwanted protein products. Myeloid cells may also be used to enhance the clearance of excess waste or diseased tissue independently via phagocytosis.

Moving forward, myeloid-based cellular therapy appears to be heading in three general directions: the enhancement of de novo immunomodulatory functions via in vitro stimulation with drugs, peptides or antigens; the insertion or removal of genetic information to enhance neuroprotection or eliminate neurodegenerative responses; or use as a “Trojan horse” for microvesicles containing therapeutic molecules, enzymes or gene vectors intended for neurons [209,210] (Figure 3). In the gene modification arena, optimized methods including lentiviral/adenoviral vectors and, most recently, customizable nucleases such as zinc finger nucleases [211] and CRIPSR-cas9 [212] have recently been developed, although it remains to be seen whether the efficiency, stability, and long-term safety of these systems are sufficient for widespread use [213]. Likewise, microvesicle and nanoparticle-loaded cell delivery is beginning to receive attention as a viable method of CNS therapeutic delivery, with initial trials indicating it is possible to target the CNS with loaded macrophages [214]. Further study will likely be needed to establish the optimal components and safe clearance of these delivery vehicles.

In summary, much progress has been made in the understanding of myeloid cell function. However, much more remains to be learned regarding the functional and associated molecular heterogeneity of myeloid subsets and their transport in and out of the brain. Current advances in neuro-immunology, genetics, and biotechnology hold great potential for the treatment of some of the most persistent and devastating illnesses affecting the CNS. We anticipate many new insights will be gained as a result of these efforts.

## Abbreviations

CNS	Central nervous system
BBB	Blood–brain barrier
CSF	Cerebrospinal fluid
ISF	Interstitial fluid
EAE	Experimental autoimmune encephalitis
CAA	Cerebral amyloid angiopathy
AD	Alzheimer’s disease
MS	Multiple sclerosis
LSD	Lysosomal storage disease
TSPO	Translocator protein (18kDa)
HSC	Hematopoietic stem cell
